# Letters from Professors J. Hughes Bennett, of Edinburgh, and Carl Rokitansky, of Vienna, on the Post-Mortem Examination of the Late S. S. Whitney

**Published:** 1859-11

**Authors:** 


### SELECTIONS.

### *Letters from Professors* J. Hughes Bennett, *of Edinburgh, and* Carl Rokitansky, *of Vienna, on the Post-Mortem Examination of the late* S. S. Whitney.

The following opinions relative to the pathological appearances found in the autopsy of the late Mr. Whitney are of great interest, in connection with the history of this remarkable case. Commenting is unnecessary. The agreement of both of these celebrated pathologists, whose opinions are here given, upon all the essential points in the case, is conclusive. To both of them was forwarded a copy of the February number of the *Monthly*, containing a full report of the proceedings of the Academy of Medicine when this subject was under its consideration. In addition to this, a copy of the original post-mortem, certified by his Honor the Mayor of New York, was sent to Prof. Rokitansky, by his former pupil and personal friend, Dr. Charles Bernacki, of this city. Dr. Bernacki has kindly placed Prof. Rokitansky's reply at our disposal. The letters are placed in the order of time in which they were received.—To the translation, here given, we append the German, that both may be seen by those who are familiar with the language in which the original is written.

### PROF. BENNETT'S LETTER.

Edinburgh, May 18, 1859.

*My Dear Doctor*—I have hesitated for some time as to how I ought to reply to your letter; that is, whether it would be better to enter into a lengthy criticism of Mr. Whitney's case, or simply answer the two queries you have put to me. After carefully studying the case itself, I find so much to comment on in the various branches of diagnosis, pathology, treatment and ethics, that I feel constrained to throw my notes aside, and give up the idea of entering into the matter fully, as it would oblige me to write a treatise rather than a letter.

You request my opinion: 1. As to the tubercular character of the pulmonary abscess. 2. As to whether such effect as Dr. Beales intimates could have been produced by the injection of nitrate of silver, which was employed.

1st. *As to the tubercular character of the pulmonary abscess.*—The description given by Dr. Green of the physical signs on the 25th October, 1858, is remarkably clear, (see *Am. Monthly*, p. 104,) and can leave no doubt as to the existence of condensation of the left lung at the apex, with softening of the tissue. When Dr. Beales was called in, Dec. 14th, he does not seem to have made any physical examination of the chest. At all events, no account of any is given from that time up to the period of the patient's death, (*Monthly*, p. 111 to p. 115.) But he tells us afterwards, that he had previously made such examination (p. 117) towards the end of October; he gives no description of these signs, but only states his opinion, viz., that "he could not discover any tubercles in his lungs, and did not believe any existed." The report of the post-mortem, however, demonstrates that the physical examination of the lung, made by Dr. Green, was in every respect correct, (*Monthly*, p. 116.) "The whole of the upper part of this lobe (the left) was red and solid—hepatized." "At the commencement of the bronchial ramifications there was an open cavity, about the size of a small black walnut, of a redish-brown color, and irregular villous surface, as though a slough had separated." This answers thoroughly for the flat sound, detected two months previously, over the upper portion of the left lung, and the humid rale audible below the left clavicle, on inspiration and expiration.

As to the nature of the disease, Dr. Beales conceives it to have been acute and recent pneumonia, (p. 118.) I cannot think so, when I consider the accurate account of physical signs given two months previously, then indicating the condensation and softening which were subsequently found. It must have been chronic pneumonia passing into gangrene, or a limited tubercular abscess accompanied by chronic pneumonia of the apex. The description given of the lesions do not enable me to say which of these it was. In fact, I consider it a matter of little importance, because a chronic exudation of the apex so readily passes into tubercle, and an old tubercular abscess is so commonly accompanied by chronic pneumonia that, in truth, it often becomes difficult, if not impossible, to say where tubercle ends and pneumonia begins. It is enough that a chronic *exudation* was there, and that, I consider, Dr. Green fully indicated by his physical signs six or seven weeks before Dr. Beales was called in.

2d. *As to whether the treatment caused the disease.*—Dr. Beales informs us that Dr. Mott told the family, after the post-mortem examination, that they had not seen any disease that might not have been produced within a week, (*Monthly*, p. 118.) According to this idea, it conld not have been the injection into the trachea which caused the pulmonary disease, as that operation was performed on the 6th of December, without causing any irritation, and he died on the 21st. Besides, the pulmonary lesion existed on the 15th October, as proved by physical signs. The injection, therefore, could not have caused that lesion. Is it not more probable that, a chronic exudation having existed at the apex, and a cavity formed, this latter perforated the pleura, causing the pleural exudation, followed by the emphysema of the cellular tissue? At what moment did this perforation take place? We are told that Mr. Whitney visited Dr. Green on the 14th of December, after breakfast, (the time is not stated;) the doctor passed an instrument into his throat, and finding some obstruction, he pushed the instrument with some force; he (Mr. W.) felt something give way, immediately experienced severe pain about the top of the windpipe, and told the doctor he had "hurt him;" he returned home, informed the family of what had occurred, and was seen by Dr. Beales at 1 P. M., (p. 112.) Doctor Green's account of what happened on the 14th is different. According to him, (*Monthly*, p. 106,) when the sponge reached the glottic opening, the patient partially closed the throat, so the instrument did not enter the windpipe at all. It was at once removed, no more force having been used than that which is constantly employed every day in operations on the air-passages. The patient, after talking a while with Dr. Foy and myself, and remarking that "the operation hurt him more," or that "he felt it more than usual," he left, with the arrangement that he should return the next day and have the tube employed. The patient's account to Dr. Beales is, that at the moment the probang was arrested at the glottis, he felt something give way, and experienced *severe* pain about the top of the windpipe, (p. 112.) But is this consistent with the fact, that he talked for a while with Drs. Foy and Green, and went home intending to come next day, &c.? Again, was the pain, if felt *at the top* of the windpipe, symptomatic of perforation of the pleura in the

2

chest? It appears to me difficult to answer these questions, more especially when we read the account of the alarming condition of Mr. W. at 1 o'clock, when first seen by Doctor Beales, (*Monthly*, p. 111.) I am therefore, inclined to ask, what occurred in the interval between the patient's leaving Dr. Green and his seeing Dr. Beales? This may have been an interval of three or four hours, and of this most important period I can find no account whatever. It is true Mr. Whitney seems to have had the impression that his severe symptoms commenced when the sponge was arrested, but this is negatived by the evidence of Drs. Green and Foy, who saw no evidence of severe pain even in the throat.—When, then, did the pulmonary and pleural perforation take place, which induced the emphysema and subsequent symptoms? With the facts at present before me, I cannot answer this question, but it is clear to my mind that it may have occurred during the hours not accounted for; that it might have been altogether spontaneous, connected only with the progress of the chronic pulmonary lesion; and that it had no relation whatever to the operation performed by Dr. Green.

In addition to the lesion already referred to, it appeared, on dissection, there was an abscess behind the larynx, and stretching towards the pharynx, the size of a hen's egg, with an opening at its upper and posterior part, large enough to admit the end of the forefinger. This abscess was not discovered before death, by any of his attendants, medical or surgical. Dr. Mott, a great authority in surgery, asserts that this was an acute abscess. Dr. Beales distinctly states, (*Monthly*, p. 119,) that in his opinion it was caused on the 14th December, by the accidental laceration of the pharynx by the probang; and his account of the symptoms, as he observed them at 1 P. M., on that day, supports the supposition, especially the intense pain in the region of the larynx, shooting through the cervical vertebræ, and down the course of the trachea to the chest; he kept grasping the larynx, &c. (p. 111.) Now, as the larynx was shown subsequently to be healthy, it is probable that these symptoms were connected with the pharyngeal abscess. But how?—Is such pain consistent with the first commencement of an abscess, or did it accompany the rupture of an abscess previously formed? By what signs was it pronounced to be acute? Might it not have existed before the operation of the 14th? Might it not have come subsequently? On all these points I will not venture to speak. But having now used the probang in many hundred cases, and under all circumstances, I am at a loss to understand how its being arrested at the glottis would lacerate the pharynx, so as to give rise to an abscess, or cause the bursting of an abscess which had already formed—"downward, behind and below the thyroid cartilage." I cannot but think that the application of the sponge had no more to do with this abscess, than it had with the exudation into the apex of the left lung. Yours, very truly and sincerely,

J. HUGHES BENNETT.

PROF. ROKITANSKY'S LETTER.

*Most Esteemed Friend*—I received your letter on the 8th, along with the documents relating to the sickness and death of S. S. Whitney, through Mr. Wallner, or rather, as I was not at home, through my wife. First permit me to express my most heartfelt thanks to you, and my trans-Atlantic friends, for the friendly remembrance which you show in the recognition of my scientific labors, and the honorable confidence reposed in me. And now to my task. I have devoted my entire attention to the documents you sent me, as you will perceive, *absque omni partium studio.*

Certainly the most important point is to determine the nature of the cavity in the left lung, "just at the root, or the commencement of the ramifications of the bronchiæ." This is very difficult, for two reasons: 1. Because the description of the cavity is very imperfect; and 2. Because the whole report (Gesammtbefund) is very defective, inasmuch as a complete dissection was not made. Neither sufficient local nor sufficient general data are given for a diagnosis.

In the last point of view, particularly, we are not certain whether a tuberculous individual or not is under consideration. It would seem as though no actual tuberculization was present.

If, under these circumstances, I give my opinion in compliance with your request, it can be of value only as a supposition.

1. The abscess in the larynx might be the result of a perichondritis; this abscess broke externally in to the contiguous circumjacent areolar tissue. The reddening of the mucous membrane of the air-passages is of no importance.

2. I do not consider it as proved that the condition of the upper lobe of the left lung was that of recent hepatization. True, there was noticed a considerable pleuritic exudation on the exterior of the lobe, but I presume that this lobe of the lung had been partially attacked with atelectasis, (obturation,) in consequence of a bronchial catarrh, and was in a state of incipient destruction, (induration.)

3. The cavity in this lung (this lobe of the lung) I would consider as a bronchial sac, or rather as a cavern (bronchial cavern,) resulting from the ulcerous destruction of such a sac.

Was this ulcerative process due to a continuous, exacerbating, catarrhal inflammation of the mucous membrane of the sac, or to a tuberculization of the mucous membrane of the same? We are obliged to accept the former solution of the question, inasmuch as the report denies the presence of any tubercles.

This cavernous opening must then be considered, since from it a destruction of both layers of the pleura proceeded, as the source of the extravasation of the inspired atmospheric air into the circumjacent areolar tissues. This, then, is my opinion with reference to the significance of the results of the post-mortem examination. As regards the operative treatment, I have not been requested, in fact, to give an opinion; still, you may perhaps like to hear that also.—It is very evident that the condition met with, i. e., the abscess in the larynx, and especially the cavity in the left lung, existed before the treatment; and I also believe that the latter (the treatment) was not the cause of the death of the patient. Your sincere friend,

ROKITANSKY.

VIENNA, June 14, '59. *Amer. Med. Monthly.*

---

1. *On the Prevention and Treatment of Mental Disorders.* By GEORGE ROBINSON, M. D., Fellow of the Royal College of Physicians of London, Fellow of the Royal Medical and Chirurgical Society, Joint Lecturer on the Practice of Medicine and Lecturer on Mental Diseases in the Newcastle-on-Tyne College of Medicine, &c.—*London,* 1859. pp. 228.

2. *A Letter to the Right Honourable the Earl of Shaftesbury on the Laws which Regulate Private Lunatic Asylums; with a Comparative View of the Process "De Lunatico Inquirendo" in England and the Law of "Interdiction" in France.* By EDWARD J. SEYMOUR, M. D., F. R. S., late Senior Physician to St. George's Hospital.—*London,* 1859. pp. 59.

3. *What shall we do with our Lunatics?* By ALFRED ECCLES, Fellow of the Royal College of Surgeons.—*London*, 1859.—pp. 16.

We welcome every additional attempt to throw light upon the mysterious wanderings of the human mind, and to remove the prejudices and fears which so interrupt and frustrate the intentions of those who show us how best to deal with a difficulty which ignorance makes a scourge; and we augur well for the future when we find so many able contributors coming forward to supply the deficiency of past years; for we cannot but be struck with the fact of how rarely the distinguished physicians whom we have been accustomed to regard as authorities in this department of medicine have appeared in the literary arena. It is encouraging to believe that the true explanation of this is, that insanity has come to be regarded more in the light of an ordinary malady amenable to ordinary treatment, and that it is not thought necessary, as it formerly was, to banish from our sight, and, if possible, from our memory, the sufferers from this grievous affliction. We hope to see the unreasonable dread of insane persons still further dissipated; and we believe that nothing is so likely to accomplish this desirable end as the correct information of the public mind upon a subject which, for the sake of all concerned, necessarily requires secrecy as regards individuals, but not mystery as regard the nature and treatment of the malady. It is much to be regretted that this secrecy operates prejudicially with the public upon those who are concerned in the treatment of the insane; and we must admit that it is the more unfair because it is observed entirely in the interest of the patients and their friends, and not of those upon whom it entails obloquy, suspicion and distrust. We may say that there is an hereditary predisposition, if not universal, at least very general, to regard all insane persons as necessarily out of the pale of society; and it is to be regretted that some psychological writers rather strengthened this view of the matter, by maintaining that there can be no degree of insanity, no partial incapacity, no limited responsibility—that, in fact, a person must either be perfectly insane or perfectly sane, and that there cannot exist any intermediate mental condition which partakes of the nature of both these states. Doctor Robinson says:

"Nothing has so much tended to confuse the study of mental disease, or to impart harshness to the treatment of the insane, as a forgetfulness of the natural construction and infirmities of the human mind. It has, until recently, been most unjustifiably assumed that a broad and unmistakeable line of demarcation existed between the sane and the insane; that the detection of insanity was therefore always a matter of facility and certainty; that the lunatic was, as it were, a creature of another world, cut off by his distemper from all sympathy or kindred associations with the rest of mankind, and that the latter were consequently justified in treating him with silent neglect, if not with actual cruelty. . . . . Is there not a certain natural range of disorder incident to every mind as regards strength, harmony, and extent of development? And do not the eccentricities, the proneness to vice and crime, the indulgence of evil passions, the follies, the vanities, the weaknesses of daily life demonstrate the universality of this inherent tendency to mental disorders? For, as the body, in the creative energies of its original conformation, in the ever varying combinations and mutations of the matter of which it is composed, in its diversified and counterbalancing functions, in the very delicacy and completeness of its arrangements, constantly engenders within itself the elements and germs of disease; even so the mind, poised in a still more sensitive balance, equally composite in its nature, and infinitely more exquisite in its sympathies, and above all, vivified and penetrated by the spiritual attributes of immortality, also carries with it in its marvelous excellences and endowments the weakness of elevation and the fragility of beauty. Philosophy and religion, therefore, alike enjoin humility and humanity in our dealings with the insane. The one forces us to admit that no man is at all moments perfectly or equally rational, and the other, in its doctrine of original sin, ever recalls to our memory the preponderating tendency of the worst and weaker parts of our mental and moral nature.

The question asked by Mr. Eccles: "What shall we do with our lunatics?" is a most important one. The social position and means of the patient will, in perhaps the majority of cases, determine how and where he shall be treated. It seems to be agreed by all, that home is not, as a rule, the place in which the disturbed mind is most likely to regain its lost balance; and that relatives, by reason of their affectionate anxiety, are not the best persons to exercise that judicious control which is required.

As, then, in the words of Dr. Seymour, "it is quite impossible that the greater number of lunatics can be treated at home," the question arises, where then? The poorest class of patients can only go to the public hospitals and county asylums, where they are most liberally provided and cared for, so that the inquiry is almost entirely limited to the upper and middle classes. The course which is least at variance with their accustomed habits, is to place them under the immediate care of a medical practitioner in his own family, and this for many cases we are disposed to regard as the best course where the means are adequate to afford suitable remuneration for such an anxious charge. Next to this, Mr. Eccles advocates the practice of placing insane patients in a private house for themselves, or in lodgings, with a proper attendant, rather than incurring the stigma which doubtless to some extent attaches to those who have been in an asylum—the association with other insane persons being, in his opinion, also objectionable and occasionally prejudicial. Dr. Seymour differs with Mr. Eccles upon this point; as a perfectly disinterested observer, being unconnected with asylums, and having for several years acted as Metropolitan Commissioner in Lunacy, his opinion is entitled to considerable weight. His great objection is, that in lodgings the patient is necessarily left very much to the mercy of servants, and that under such circumstances there are too often opportunities for improper treatment, which cannot happen without being known in a licensed house.—We are disposed to agree with Dr. Seymour, and differ with Mr. Eccles, as to the effect of association in the majority of cases of insanity, for it must not be forgotten that a most essential part of the treatment of insanity consists in presenting to the disordered mind a succession of new ideas in the least exciting form, for the purpose of displacing those which have been gaining prominence through a prolonged period of neglected self-discipline; the order and regular habits of a well managed asylum are, perhaps, better calculated to carry out this treatment than the luxurious freedom, advocated by Mr. Eccles, which, though doubtless applicable to a certain class of cases of a mild form, and where there are abundant means, is wanting in what we believe to be of paramount importance in the management of a disordered mind—viz., efficient control—whilst it is only the wealthy who can meet the necessary expense.

"The expense," Dr. Seymour says, "of a private dwelling medical attendance, and personal attendance, beyond a very short period, is so very great, that only a very few families can afford it, and at length they are obliged to have recourse to a licensed house. In every view, then, of fortune, convenience, or necessity, the great majority of persons of moderate fortune, thus afflicted, must have recourse to a licensed house; the great object, then, surely, is to make the licensed houses—the necessary retreat of the larger number of the afflicted—as perfect as possible. Still, the feeling fostered by novel writers (who never, as far as I know, *really* depict a lunatic case)—the feeling for absolute secrecy which pervades society, the idea that when there is secrecy there is opportunity for injustice—all these operate on the public mind to decry similar institutions."

After deprecating the unreasoning outcry and hostility manifested against "those who undertake the ungrateful duty of passing a large portion of their lives with these examples of suffering humanity," Dr. Seymour goes on to express his opinion that the law as it exists is simply sufficient, if the working staff of the Commissioner is increased. Dr. Robinson thinks,

"That the present law of lunacy, and the administrative machinery employed under it, greatly need improvement . . . . and that in any future legislation, the utmost care should be taken to approach this difficult subject in a calm, conscientious spirit, and one utterly removed from passion or prejudice; for it needs the utmost power of intellect and clearness of moral perception to reconcile, in a law of lunacy, the conflicting claims of personal and public interests, to preserve, at the same time, the liberty of the subject and the security of society, and to ensure the humane and kind treatment of the lunatic without denying to the persons entrusted with his charge the ordinary protection afforded by English law to English subjects."

Mr. Eccles agrees with Dr. Seymour, that the present law, with some small modification, would be amply sufficient, though he differs with him as to the expediency of any considerable addition to the working staff of the Commissioner. His views are thus stated:

"I trust Parliament will not be led by the parade of a few exceptional cases to legislate hastily. The present law of lunacy, consolidated with some small modifications, would be amply sufficient to protect the lunatic if it were rigidly enforced. Its efficiency, or the reverse, is entirely in the hands of the executive. I do not think the *large* increase of the Commission, or harrassing patients with constant, visits by ever-changing officials and doctors, desirable. I would rather take stringent measures to secure that *none but persons of character and skill should be allowed to take charge of the lunatics*—would make it necessary that the certificate should bear the signature of one public medical officer, deputed to examine such cases, and of one private practitioner, and that within one month the patient should be visited by a commissioner, who should countersign the certificate or institute a formal inquiry, and should report on the accommodation, treatment, &c."

It appears, then, from the opinion of those not connected with asylums, though practicing in lunacy, that there is but little wanting in the way of change of the existing laws, but it is hinted that the working of them is susceptible of some improvement. It certainly does appear, also, that in considering the claims of this most unfortunate class, there is a tendency to overlook the rights of those whose lives, and frequently whose fortunes, are devoted to their care and treatment; and against this section of our profession there has been an outcry which all the charges substantiated against them do not seem to justify. If the requirements of society necessitate the existence of licensed houses, it would be impolitic on the part of the legislature and the executive to make the position of the proprietors in any respect more irksome than the nature of their anxious duties renders inevitable; at the same time, society has a right to expect that its afflicted members should not be made to suffer from the interested motives of the persons to whose care they are committed; it is desirable, therefore, that the responsibility of their detention should not rest upon those who have charge of them, but upon the Commissioners in Lunacy, or other properly constituted and independent authority. If the protection of two medical certificates are not sufficient, by all means increase the protection, and give the public every possible guarantee that no person shall be confined as a lunatic who is not of unsound mind, and whose malady does not imperatively call for judicious control and treatment.

It would lead us beyond our limits to go into the question as to what amount of mental infirmity shall justify interference with the liberty of the subject, but we should have a care lest our jealousy of any infringement of personal liberty should lead us to sacrifice the welfare of the individual and prejudice the interests of society, by withholding the proper remedial means, whatever they may be. It is agreed on all hands that the successful treatment of insanity is in the direct ratio of its early adoption, and that this exercises a far greater influence on the result than the apparent intensity of the symptoms as they are manifested in the early stages of the malady. If, then, it is clear that the mental manifestations are becoming more and more disordered, and the belief is unquestioned that delays can only diminish the probabilities of cure, we cannot surely be too prompt in our endeavors to rescue a fellow-creature from the most severe affliction to which intelligent beings are liable, nor should we be thrown off our guard by the apparent reasonableness of the patient's conduct and conversation on particular subjects, for it must be remembered that the most notorious of the class called criminal lunatics are those whom society had neglected, if not refused to consider insane, until the world was startled by some frightful act of atrocity which cleared up all doubts as to their mental condition, when, alas! it was too late. Bearing in mind the greatly increased prospect of cure when early treatment is adopted, it is worthy of consideration whether the patient and his family are not less likely to be prejudiced by the imputation of insanity than by the neglect of proper precautions and management in the first stage of the malady. But the refusal to recognize the evidence of insanity is, as Dr. Seymour remarks, "the paramount feeling of mankind, whether in the rich or poor," and in his experience "there is no asseveration too strong, no trick too great, to hide even from the medical adviser that others in the family besides the patient have experienced this calamity." How, then, can we hope to remove a prejudice whose strength lies in the weakness of human nature? It can only be by diffusing more correct knowledge on the subject, that we can hope to dispel the mysterious awe with which the world regards this particular malady in itself; we must show that it is susceptible of amelioration and palliation, and in the majority of cases of as perfect a cure, as any other bodily ailment, if not too long neglected. With reference to the curability of insanity, which is abundantly shown by statistics, Dr. Robinson says:

"My own experience is wholly in favor of the idea, that in the great majority of cases insanity is directly or indirectly the effect of sources of mental disturbance originating in the will or feelings of the persons affected, and which may therefore be considered as moral in their nature. The mental affection itself may not be immediately produced by the moral disturbing cause, but if the latter excite in the system a series of disordered actions terminating in the former, and which would not otherwise have been in operation, we are surely warranted in regarding it as essentially productive of the attack of insanity. Moreover, in proving that the great majority of cases of insanity arise from moral causes, we prove that it is in general a preventible disease. Many physical agencies and bodily infirmities cannot be altogether prevented. But, inasmuch as sound religion and sound philosophy alike enjoin constant warfare against the tendencies naturally existing in mankind, we are justified in believing that mental disorders, arising from those moral weaknesses, are by no means necessary and inevitable afflictions. It is no part of man's duty to bow down tamely to miseries of his own creation, and allow the light of his reason, and the best and holiest of his spiritual attributes to be polluted by the offspring of his own vices and infirmities, and when educated communities can once be made to understand and feel that the preservation of their mental health is to a very great extent in their own hands, we may expect many of the most fertile sources of mental disorder to be speedily and effectually checked."

In one important point Dr. Robinson differs with almost all the recent writers on insanity, including Esquirel, Pritchard, &c., who have maintained the reality of a morbid condition, to which the designation "Moral Insanity" has been given, and which, in Dr. Pritchard's words, is characterized by "a morbid perversion of the feelings, affections, and active powers, without any illusion or erroneous conviction impressed upon the understanding; it sometimes coexists with an apparently unimpaired state of the intellectual faculties." Dr. Robinson does not deny that the Moral Sense and Feelings in the partially insane are frequently impaired, but argues that there are few in whom the powers of the Will, the Reason, and the Conscience are so far undermined as to render them altogether incapable of self-control, and consequently irresponsible. This is no doubt perfectly true, but the advocates of the doctrine of Moral Insanity do not contend, as Dr. Robinson seems to think, that this condition, if ever so satisfactorily shown to exist, should be admitted as a bar to *all* punishment. No doubt the doctrine has been carried too far, and Dr. Robinson has some reason for saying that—

"This assumption of the possible existence of 'irresistible' and 'uncontrollable' impulses in persons admitted to be intellectually sane, is the root of all the difficulties surrounding the subject."

And we agree with him that—

"The only effective and legitimate mode of reconciling the requirements of social security with the exculpatory recognition of human weakness and man's natural proneness to crime, consists in the mitigation of our criminal laws, and the substitution of the reformatory for the retaliatory principle, as the ground of the punishments inflicted by them."

But Dr. Robinson recognizes the fact, that there are large numbers of persons of unaffected intellect in whom the governing power of the mind is so far enfeebled, that they are "habitually incapable of resisting criminal impulses or vicious cravings," and this surely amounts to something very like "irresistible" impulse. He is so well aware that this Moral Weakness is a fertile source of misery, that he believes, in these cases, the general interest of the community to be more involved, than in those of individuals "morbidly" affected. We are inclined to think that Dr. Robinson's unwillingness to regard Moral Weakness as a *morbid* condition, depends very much upon his fear that it should be confounded with natural depravity and vice, and admitted as a bar to punishment when pleaded in extenuation of crime.—We think there are few impulses which are altogether irresistible, if there be a sufficiently powerful motive to exercise control over the actions, and, therefore, few who can properly be said to be irresponsible; but we think few will deny that the responsibility attaching to individuals differ in degree, and that in many it is of a very limited kind by reason of Mental Weakness, the result of morbid action in these cases. We would on no account contend for immunity, but if punishment is inflicted, it ought surely to be modified in proportion to the presumed incapacity to resist criminal impulses.

The question as to the increase of insanity is one upon which authorities differ, and upon which it is very difficult to form an opinion. Statistics are quoted both to prove and disprove the position, and it is maintained with considerable show of reason, that the apparent increase is in a great measure, if not altogether, owing to the fact that more cases are brought under observation, and more attention is paid to them; but making allowance for these, the weight of evidence seems to be in favor of the opinion that there is a certain increase in the number of insane persons beyond what could be referred to the increasing proportion of the population. The chief cause of this is doubtless to be found in the wear and tear of the great battle of life, too often carried on without regard to any consideration but the gratification of an ever restless ambition, which leads men of every rank and degree to sacrifice present happiness in the fierce struggle for wealth and pre-eminence. We cannot but fear that the increasing civilization of the age of which we constantly hear such complacent commendation, is not without serious drawbacks, and that society pays for its advantages by the greater selfishness of its members, and by the prevalence of a less lofty tone of morality. Dr. Robinson has expressed his views on this subject as follows:

"Notwithstanding, then, the enormous intellectual advances made during the present age, there probably never was a period in the history of this country when happiness and contentment were less generally diffused throughout the different classes of society. The increasing tendency to the concentration of wealth in the hands of a few, necessarily produces discontent among the many; while the incessant striving after social elevation, as one of the results of the more general equalization of education, the intensity of competition, the love of display, so unnatural to the old English character, the introduction of many foreign vices, and the frequent faithlessness of men holding important trusts, have all united to engender a state of feeling in the highest degree injurious to the mental condition of the community. And in investigating the moral causes of insanity, we shall discover ample evidence of its frequent origin in the vices of a spurious and hollow civilization."

We have derived much pleasure from the perusal of Dr. Robinson's treatise, which is evidently the production of a philosophic mind, and we trust that it will be extensively read, believing that his views are generally sound, and knowing that his experience has been considerable. The suggestions also of Dr. Seymour and Mr. Eccles, are well worthy of consideration, at a time when Parliament needs to be guided by the unprejudiced opinions of experienced men in legislating upon a very difficult subject, under the influence of a sort of panic created by exaggerated statements, and increased by the fears which have their origin in a very imperfect knowledge of the subject on the part of the public. —*British and Foreign Medico-Chirurgical Review.*

